# The Whole Side of It—An Interview with Neil Risch

**DOI:** 10.1371/journal.pgen.0010014

**Published:** 2005-07-25

**Authors:** Jane Gitschier

Neil Risch is the Lamond Distinguished Professor in Human Genetics and Director of the Center for Human Genetics at the University of California, San Francisco, California, United States. He has held faculty appointments at Columbia, Yale, and Stanford Universities.

Trying to track down Neil Risch is the stuff of legend. E-mails to him can bounce back with the comment line “overwhelmed by email.” Phone calls lead to voicemail, and faxes to a tepid response from his assistant. It's not that he's reclusive or off windsurfing, he's simply a whirlwind of genetics ideas and activity.

Indeed, when the University of California at San Francisco was looking for a director of the new Center for Human Genetics, Neil's name quickly came to the top of the heap. With wide-ranging experience and interests, he was described by one of the field's founding fathers as “*the* statistical geneticist of our time.” It didn't hurt that he is a mensch.

I managed to trap Neil in his bright new office on the ninth floor of the west tower off Parnassus Avenue. Still spartan, with only a computer, a phone, and a chair, the office's view spanned Golden Gate Park, the Marin headlands, and the Pacific Ocean. The vista was interrupted only by the jarring copper-clad tower of the new museum under construction in Golden Gate Park. It was a brilliant blue, warm afternoon, and I looked forward to spending some one-on-one time with this man, with his infectious laugh and his intellectual stamina.

**Jane Gitschier:** Let's start with the broad view. What really interests you?

**Neil Risch:** My passion, really, is the interplay between population genetics and clinical applications—to see the whole side of it.

[Fig pgen-0010014-g001]When I was in graduate school, I came out of math. Three weeks into my first course in human genetics [as part of a new biomathematics graduate program at the University of California at Los Angeles], I knew that it was what I wanted to do. One, I loved the subject matter. Two, I loved the quantitative aspect of it. Three, I loved the intuitive aspect of it. It was almost like I could predict the next lecture. It was a perfect fit for me. My other passion was population genetics, but there weren't many career opportunities in that field back then. So always there was this latent passion for population genetics without the opportunity to act on it.

**Figure pgen-0010014-g001:**
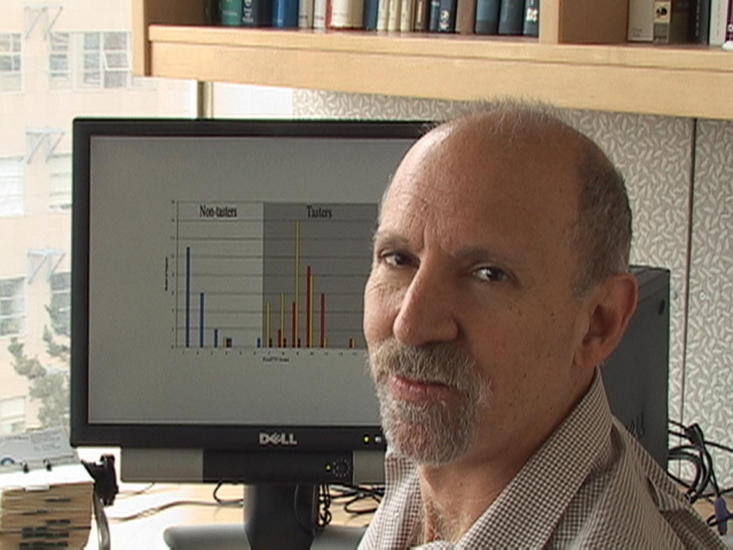
**Neil Risch**

The thing that has been exciting for me is that I saw ten years ago where the field was going. Having the sequence of the human genome provides the opportunity to look at the variation in that sequence as well, which leads to the marriage of several areas, but particularly human population genetics with disease studies—genetic epidemiology.

But the fields have never been so intimately related as they are now, and I am just thrilled. I get to marry the two things I love to do. In the old days, the NIH [National Institutes of Health] would never fund a study in population genetics, but now it does because you need to understand human population history and genetics to undertake all these studies of human genetic variation underlying disease susceptibility.

**Gitschier:** So many people want to collaborate with you. How do you choose what projects to become involved with? Did you initiate most of the projects you work on, or do people come to you?

**Risch:** In my Stern Award address [presented to the American Society of Human Genetics in 2004], I talked about the population genetics analogy of selection and drift. Some things are planned and have a natural scheme to them, and some things are just random. Some things I have become interested in and I have initiated, and other things have come to me and I've participated in them either because it was appropriate, given the setting where I was working, or I was interested in the project and wanted to have a collegial relationship.

There has been quite a range of the projects I have gotten involved in, especially on the clinical side. I think it's been good for me that I haven't focused on one particular area, like cancer or psychiatry. Some people could say, “He's like a dilettante,” but I think it has given me a broad perspective on the clinical application and the commonalities in terms of the issues involved across diseases, and some of the unique aspects. I value the fact that I've been able to be involved in a lot of different things. I feel incredibly fortunate to have become established in a field so that I've had a lot of opportunities in terms of different people approaching me for collaborations. It's been great.

**Gitschier:** You are an Ashkenazi Jew and belong to a conservative temple here in San Francisco. You also are well known for your work on diseases, such as torsion dystonia, that affect this particular population. Do you think you gravitate to these problems because of your ethnicity?

**Risch:** Early on, I was interested in population genetics and I knew about this debate about the presence of the lysosomal storage diseases in the Jews. Why did the Jews have all these diseases?

**Gitschier:** I just assumed it was selective advantage. What was the debate?

**Risch:** Between selection and drift—as it always is.

This actually started with the dystonia work. At the time, there was a raging question about the mode of inheritance. They knew it [severe early-onset idiopathic torsion dystonia] was more prevalent in Ashkenazi Jews. Some people thought it was dominant, but there was a major paper that said it was recessive just like all other major Ashkenazi diseases.

And I was interested. It was a nice statistical problem, and it was in the Jewish population, which I was interested in both for scientific and historical reasons, and because of my own identity.

So we did a study. Susan Bressman, my colleague at Columbia, systematically went out and clinically examined all the first- and second-degree relatives of all the early-onset Jewish cases—parents, siblings, children, nieces and nephews, half-sibs, uncles and aunts, maybe grandparents. We analyzed the data, and the rates of dystonia in the relatives were the same in all the first-degree relatives; the siblings did not have a higher risk. About 15% for everybody, across the board. We concluded that it was autosomal dominant with low penetrance [30%].

We did a formal segregation analysis. We could clearly, overwhelmingly reject a recessive model. And at the time I thought, “Well, this is fascinating!” because we had a dominant disease with a founder effect in the Jewish population. And we even suggested that this would be valuable for gene mapping—by linkage disequilibrium [LD] analysis. And after mapping the disease, we found strong LD right away. So this was very clear evidence that we were dealing with a relatively recent founder mutation.

I know this is a long story, but it was through that project that I got interested in Jewish population genetics.

**Gitschier:** It seems as though every time I open the science section of the *New York Times,* you are featured in it. These articles, at least lately, focus on your adherence to an often politically incorrect idea, such as the genetic basis for race or the way NIH should spend its money on diseases of addiction. Do you deliberately choose controversy?

**Risch:** I think historically I have avoided it. Perhaps this is what job security offers you—the opportunity to get involved in potentially more controversial questions. And I think I've decided that playing it safe is not the way to go. I just don't believe that anymore. These are big important subjects and I just don't think they should be avoided.

**Gitschier:** Let's talk about the former, the genetic basis of race. As you know, I went to a session for the press at the ASHG [American Society for Human Genetics] meeting in Toronto, and the first words out of the mouth of the first speaker were “Genome variation research does not support the existence of human races.”

**Risch:** What is your definition of races? If you define it a certain way, maybe that's a valid statement. There is obviously still disagreement.

**Gitschier:** But how can there still be disagreement?

**Risch:** Scientists always disagree! A lot of the problem is terminology. I'm not even sure what race means, people use it in many different ways.

In our own studies, to avoid coming up with our own definition of race, we tend to use the definition others have employed, for example, the US census definition of race. There is also the concept of the major geographical structuring that exists in human populations—continental divisions—which has led to genetic differentiation. But if you expect absolute precision in any of these definitions, you can undermine any definitional system. Any category you come up with is going to be imperfect, but that doesn't preclude you from using it or the fact that it has utility.

We talk about the prejudicial aspect of this. If you demand that kind of accuracy, then one could make the same arguments about sex and age!

You'll like this. In a recent study, when we looked at the correlation between genetic structure [based on microsatellite markers] versus self-description, we found 99.9% concordance between the two. We actually had a higher discordance rate between self-reported sex and markers on the X chromosome! So you could argue that sex is also a problematic category. And there are differences between sex and gender; self-identification may not be correlated with biology perfectly. And there is sexism. And you can talk about age the same way. A person's chronological age does not correspond perfectly with his biological age for a variety of reasons, both inherited and non-inherited. Perhaps just using someone's actual birth year is not a very good way of measuring age. Does that mean we should throw it out? No. Also, there is ageism—prejudice related to age in our society. A lot of these arguments, which have a political or social aspect to them, can be made about all categories, not just the race/ethnicity one.

**Gitschier:** I have heard you say, “Don't politicize the human genome.”

**Risch:** I have a strong problem with the way politicians use this information. [Former President] Clinton, for example, when the first draft of the human genome sequence came out, made a statement about how all people in the world, in terms of their genetic makeup, are 99.9% the same. His intent—to reduce conflict among peoples—is noble. People on the left, anthropologists and sociologists, do the same thing. They use the 99.9% figure as an argument for social equality. But the truth is that people do differ by that remaining 0.1% and that people do cluster according to their ancestry. The problem is that others could use that information to create division.

**Gitschier:** Do you ever feel that the press misrepresents you?

**Risch:** Don't we all feel that to some extent? They always take the simple side, which leads to misinterpretation. So there are risks in talking to the press, especially on controversial subjects.

**Gitschier:** You have a brother who is an academic, and at one point you and he were both on the faculty at Yale. Is that genetic? Tell us about the environment that you grew up in that ultimately led to producing two academicians. I don't know what your brother's field is.

**Risch:** So interesting. You tell me, is this “nature” or “nurture”?

My brother, my only sibling [Harvey Risch], is 20 months older than I. Mathematics was his skill set also. He went to Cal Tech, starting a year before me—both of us math majors. Then he decided to go to medical school, so he did additional coursework, and we graduated simultaneously. He went to UCSD to medical school, and had to do a thesis—so he came to UCLA in my department and lived with me. Then he applied to do a PhD in biomathematics in my department [but went to the University of Chicago instead]. His first paper and my first paper appeared in the same issue of *Annals of Human Genetics,* and we didn't even know it. Then Harvey decided to do epidemiology as a post-doc, while I was learning epidemiology at Columbia. Our grades, SAT scores, GRE scores—everything pretty much the same.

**Gitschier:** Sounds like the premise for a simple quantitative analysis. Do you think you are both hardwired to do mathematical problems? Or did your family just sit around at dinner doing math problems?

**Risch:** Not at all. My mom [Sonia Risch] was very artistic and intuitive. Great artist, writer, actress—and brilliant at all of it. My father was a clinical psychiatrist. But if you look at my family history, on my mother's side there are a lot of MDs and on my father's side there is a lot of math. So if you want to make a genetic hypothesis here, my brother is the confluence of both, maybe, and maybe me, too. My brother and I would talk about stuff a lot. My mother would say, “Oh, they're talking Fortran.”

“My passion, really, is the interplay between population genetics and clinical applications—to see the whole side of it.”

**Gitschier:** You have been working with the epidemiologists at Kaiser Permanente [a health-care provider] in Oakland for the past seven years. I understand you spend every Wednesday there. How did this collaboration come about, and what is it you are trying to accomplish? Other than numbers, is there something the Kaiser resource can do that say, Iceland, couldn't?

**Risch:** Can it ever!

**Gitschier:** Why don't you tell me what's so cool about it?

**Risch:** When I went to Stanford, at the back of my mind was this issue about Kaiser. This comes from my epidemiology background. This is the advantage of being multidisciplinary. If I could push anything, it's the value of seeing the links between various disciplines and marrying them. One, Kaiser's membership is a cohort—you don't have to construct it from scratch. And it's followed over time, for many people over 20 years. Two, they have computerized databases where every contact a patient has with the health-care system, every inpatient, outpatient, and pharmacy visit, every visit with doctors outside the Kaiser system gets recorded, every X ray, every lab test, basic biochemistries—all computerized. Three, it is the health-care provider for one-third of the Bay Area and a very good representation of Bay Area population. It is missing only the very high and very low end of the socioeconomic ladder. All major ethnic groups are represented. To me, it's the most wonderful laboratory for doing population genetic and genetic epidemiologic research.

As you can tell, I'm a very strong believer of inclusion of a variety of people of varying racial/ethnic backgrounds in research. There is everything to be gained from doing so—not just politically, but scientifically. Also, ethically it's the right thing to do. And I'm concerned in this whole discussion that people may be scared off from the genetics research and that's the battle.

One big issue that I think will go a long way towards addressing this problem is to do everything we can to recruit more minority scientists to human genetics. For the research to have credibility in minority communities, there must be representation from those communities among scientists. And I want to be involved in that process also.

**Gitschier:** In Iceland, you've got all the ancestry data, and so you can do traditional linkage analysis, but in Kaiser, you're going to do association studies.

**Risch:** That's right. Because of the way the technology is moving, this is a tremendous resource for doing that.

**Gitschier:** This is a very long-term study. There must be an element of “Oh, I see this gelling” and it just can't go fast enough.

**Risch:** You're right, it's been a long process. There are complexities and financial issues.

This is really the best opportunity we have in the United States to do something along these lines, and it's been a little frustrating that it's been difficult to get the support and funding for it, but I'm patient. Because when you believe in something and know it's right, you have the patience to see it though. 

